# Alterations of Phagocytic Activity and Capacity in Granulocytes and Monocytes Depend on the Pathogen Strain in Porcine Polytrauma

**DOI:** 10.3389/fmed.2021.645589

**Published:** 2021-04-06

**Authors:** Jan Tilmann Vollrath, Felix Klingebiel, Felix Marius Bläsius, Johannes Greven, Eftychios Bolierakis, Andrea Janicova, Ildiko Rita Dunay, Frank Hildebrand, Ingo Marzi, Borna Relja

**Affiliations:** ^1^Department of Trauma, Hand and Reconstructive Surgery, Goethe University, Frankfurt, Germany; ^2^Department of Trauma, University of Zurich, Universitätsspital Zurich, Zurich, Switzerland; ^3^Experimental Radiology, Department of Radiology and Nuclear Medicine, Otto von Guericke University, Magdeburg, Germany; ^4^Department of Trauma and Reconstructive Surgery, RWTH Aachen University, Aachen, Germany; ^5^Institute of Inflammation and Neurodegeneration, Otto von Guericke University Magdeburg, Magdeburg, Germany

**Keywords:** phagocytes, inflammation, infection, trauma, pathogen

## Abstract

**Background:** Polytraumatized patients undergo a strong immunological stress upon insult. Phagocytes (granulocytes and monocytes) play a substantial role in immunological defense against bacteria, fungi and yeast, and in the clearance of cellular debris after tissue injury. We have reported a reduced monocytes phagocytic activity early after porcine polytrauma before. However, it is unknown if both phagocyte types undergo those functional alterations, and if there is a pathogen-specific phagocytic behavior. We characterized the phagocytic activity and capacity of granulocytes and monocytes after polytrauma.

**Methods:** Eight pigs (*Sus scrofa*) underwent polytrauma consisting of lung contusion, liver laceration, tibial fracture and hemorrhagic shock with fluid resuscitation and fracture fixation with external fixator. Intensive care treatment including mechanical ventilation for 72 h followed. Phagocytic activity and capacity were investigated using an *in vitro ex vivo* whole blood stimulation phagocytosis assays before trauma, after surgery, 24, 48, and 72 h after trauma. Blood samples were stimulated with Phorbol-12-myristate-13-acetate and incubated with FITC-labeled *E. coli, S. aureus* or *S. cerevisiae* for phagocytosis assessment by flow cytometry.

**Results:** Early polytrauma-induced significant increase of granulocytes and monocytes declined to baseline values within 24 h. Percentage of *E. coli*-phagocytizing granulocytes significantly decreased after polytrauma and during further intensive care treatment, while their capacity significantly increased. Interestingly, both granulocytic phagocytic activity and capacity of *S. aureus* significantly decreased after trauma, although a recovery was observed after 24 h and yet was followed by another decrease. The percentage of *S. cerevisiae*-phagocytizing granulocytes significantly increased after 24 h, while their impaired capacity after surgery and 72 h later was detected. Monocytic *E. coli*-phagocytizing percentage did not change, while their capacity increased after 24–72 h. After a significant decrease in *S. aureus*-phagocytizing monocytes after surgery, a significant increase after 24 and 48 h was observed without capacity alterations. No significant changes in *S. cerevisiae*-phagocytizing monocytes occurred, but their capacity dropped 48 and 72 h.

**Conclusion:** Phagocytic activity and capacity of granulocytes and monocytes follow a different pattern and significantly change within 72 h after polytrauma. Both phagocytic activity and capacity show significantly different alterations depending on the pathogen strain, thus potentially indicating at certain and possibly more relevant infection causes after polytrauma.

## Introduction

Trauma is responsible for around 5 million deaths per year worldwide with more than a quarter (29%) of these deaths following road traffic injuries (1). Polytraumatized patients surviving the initial phase after injury are at great risk to die from late-occurring complications like sepsis, lung failure or multi organ failure (MOF) (2, 3). Although clinical management of polytraumatized patients has significantly improved over the past decades leading to a reduced mortality, these advances are somewhat mitigated when the trauma patient succumbs to postinjury sepsis, which increases mortality from 7.6 to 23.1% (4–7). Polytraumatized patients are at risk of infections at least partly due to the disruption of immune system homeostasis (7). Thus, it is critical to investigate immunological changes after trauma to identify patients at risk for posttraumatic infections as well as possible therapeutic targets. Neutrophilic granulocytes are the first line of defense against rapidly dividing bacteria, fungi, and yeast and, thus, are instrumental in promoting resistance to infection (8). Elimination of pathogens by neutrophils can be achieved either by phagocytosis and subsequent killing using reactive oxygen species or antibacterial proteins (cathepsins, defensins, lactoferrin and lysozyme), or by degranulation of antibacterial proteins into the extracellular milieu and/or by NETosis (9, 10). Monocytes as another major type of circulating phagocytes eventually migrate into damaged or traumatized tissues where they accomplish their terminal differentiation into macrophages (11). Monocytes/macrophages contribute substantially to the adaptive immunity *via* antigen presentation to T cells and a general orchestration of the immune reactions (11).

The most frequent nosocomial infections of the lower respiratory tract (28%), the urinary tract (24%) or surgical wound infections (18%) after polytrauma are often caused by *S. aureus* and *E. coli* (12, 13). When the human body is confronted with invasive pathogens such as bacteria or fungi, these can be cleared from infection sites by professional phagocytes like monocytes and granulocytes (14). Phagocytosis thereby is an important contributor to the first line of defense against infections (14). With regard to development of infections during the clinical course after trauma divergent results on phagocytic activity of circulating neutrophils and monocytes have been reported (15–17). Spittler et al. compared the phagocytic capacity of monocytes from patients with sepsis with low or high IL-6 serum concentrations and observed significantly increased phagocytic properties as well as worse outcomes in patients with high IL-6 levels (17). Reduced phagocytic activity of granulocytes and monocytes has been reported in septic patients after trauma or surgery and reduced phagocytic activity of neutrophils in the first 24 h of sepsis has been a negative predictor for survival (15, 16). In our study group we investigated the phagocytic activity of granulocytes and monocytes after severe trauma and observed early decreased activity of granulocytes followed by significant increase from day 2 till the end of the study period (18). Phagocytic activity of monocytes returned to values comparable to healthy volunteers after 2 days (18). Furthermore, we observed decreased phagocytic activity and capability of monocytes for *S. aureus* early after trauma in the porcine polytrauma model (19). The porcine polytrauma model offers great opportunity to study posttraumatic immunological changes under standardized conditions. Thus, we investigated the phagocytic activity and capacity of granulocytes and monocytes for three different pathogen strains in order to further characterize immunological changes after polytrauma, and to potentially get closer to answering the urgent question what mechanisms put patients at risk for developing infectious complications after polytrauma.

## Materials and Methods

### Ethics

All experiments were performed in compliance with the federal German law with regards to the protection of animals, institutional guidelines and the criteria in “Guide for the Care and Use of Laboratory Animals” (20). During the whole study animals were consistently handled in accordance with the ARRIVE guidelines (21) and experiments were authorized by the responsible government authority (“Landesamt für Natur-, Umwelt- und Verbraucherschutz”: LANUV-NRW, Germany, AZ: 81.02.04.2018.A113). All Animal experiments were performed at the Institute of Laboratory Animal Science & Experimental Surgery, RWTH Aachen University, Germany.

### Animals

A total of eight male German landrace pigs (*Sus scrofa*, 3-month-old, 30 ± 5 kg) from a disease-free barrier breeding facility were included in this study. Prior to experimentation the animals were fasted over night with free access to water. All animals were housed in air-conditioned rooms, allowed to acclimatize to their surroundings for at least 7 days before surgery and underwent examination by a veterinarian before experimentation. The large animal polytrauma model has been described before (22).

### Experimental Model

**Figure 1:**
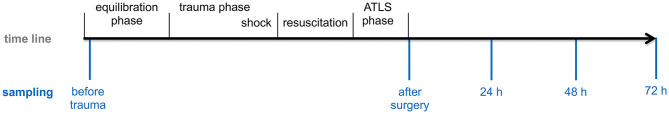
Experimental design. Pigs underwent polytrauma consisting of lung contusion, liver laceration, tibial fracture and hemorrhagic shock followed by fluid resuscitation and fracture fixation with external fixator. Blood sampling was performed at the beginning after implementation of the central venous catheter (before trauma), after ATLS phase (after surgery) and after 24, 48 and 72 h.

Eight animals underwent polytrauma (PT) with a standardized tibia fracture, liver laceration, unilateral blunt chest trauma, hemorrhagic shock (40 ± 5 mm Hg for 90 min), ensuing resuscitation and surgical fracture fixation via external fixation. Before experimentation all animals were pre-medicated with an intramuscular application of azaperone (Stressni™, Janssen, Germany) in a dose of 4 mg/kg. Prophylactic antibiotic treatment with cefuroxime 1.5 g i.v. (Fresenius Kabi, Bad Homburg, Germany) was administered before surgery and every 24 h for the whole experiment. Anesthesia was induced with an intravenous injection of propofol (3 mg/kg, Propofol Claris 2% MCT, Pharmore GmbH, Ibbenbüren, Germany) followed by an orotracheal intubation (7.5 ch tube, Hi-Lo Lanz™). During the whole study period of 72 h general anesthesia was maintained with intravenous injection of propofol, fentanyl (Rotexmedica, Trittau, Germany) and midazolam (Rotexmedica, Trittau, Germany) at a sufficient level in order to prevent any pain or awareness. The animals were ventilated with volume control mode (Draeger, Evita 4, Lübeck, Germany) at a tidal volume setting of 6–8 mL/kg, positive end expiratory pressure (PEEP) of 8 mm Hg (plateau pressure < 28 mm Hg) and a pCO_2_ of 35–45 mm Hg. Central venous catheter (4-Lumen Catheter, 8,5 Fr, ArrowCatheter, Teleflex Medical, Germany) was aseptically inserted into the right external jugular vein for anesthesia and fluid administration and continuous monitoring of the central venous pressure. In order to induce hemorrhage a two-lumen hemodialysis catheter (Arrow International, Teleflex Medical, Germany) was aseptically inserted into the left femoral vein. An arterial pulse contour cardiac output (PiCCO, Pulsion Medical Systems, Germany) catheter was aseptically inserted into the right femoral artery to monitor blood pressure and for blood gas analysis. A urinary catheter was inserted into the bladder (12.0 Fr, Cystofix, Braun, Melsungen, Germany). The baseline measurements were acquired after an equilibration period of 1 h. The polytrauma was induced as previously described with slight modifications (22). Before trauma induction the fraction of inspired oxygen (FiO_2_) was set to 21% as this depicts the conditions during trauma more accurately. Fluid administration was reduced to 10 mL/h. Warming with forced-air warming systems to maintain normothermia was stopped and animals were allowed to descend into hypothermic state after hemorrhagic shock as this mimics the preclinical scenario. After placing the animal on the left side, tibial fracture was induced using a bolt gun (Blitz-Kerner, turbocut JOBB GmbH, Germany; ammunition: 9 × 17, RUAG Ammotec GmbH, Fürth, Germany). After that animals were placed on the right side and blunt thoracic trauma was induced with a bolt shot to the left dorsal lower thorax. Then a midline-laparotomy and uncontrolled bleeding for 30 s after crosswise incision of the caudal lobe of the liver (4.5 × 4.5 cm) was induced followed by packing with five sterile gauze-compresses (10 × 10 cm). Hemorrhagic shock was induced via exsanguination via the femoral venous catheter until a mean arterial blood pressure (MAP) of 40 ± 5 mm Hg was reached and maintained for 90 min. Resuscitation was managed by adjusting the FiO_2_ to baseline again and re-infusing the withdrawn blood and additional crystalloid fluids (4 mL/kg body weight/h). Rewarming was performed using forced-air warming system until normothermia was reached (38.7–39.8°C). After induction of trauma and resuscitation clinical treatment of the tibia fracture was performed using external fixation according to established trauma guidelines. The intensive care treatment as well as the management of complications followed the standardized clinical protocols according to the latest recommendations of the European Resuscitation Council and the Advanced Trauma Life Support (ATLS®) (23, 24). After 72 h animals were euthanized using potassium chloride. The experimental design is shown in Figure 1.

### Blood Sampling and Processing

Blood samples for *ex vivo in vitro* phagocytosis assay were obtained immediately after implementation of the central venous catheter (before trauma), after surgery and after 24, 48, and 72 h in heparin tubes (S-Monovette® Lithium-Heparin, Sarstedt, Nümbrecht, Germany), kept at room temperature and immediately processed for further analysis via FACS.

### *Ex vivo in vitro* Whole Blood Stimulation Phagocytosis Assay

Blood samples (200 μL) were transferred to polystyrene FACS tubes (BD Biosciences, Franklin Lakes, USA) and 5 μL *phorbol 12-myristate 13-acetate* (PMA, Merck, Darmstadt, Germany) with a final PMA concentration of 0.1 μg/mL were added to the samples followed by incubation at 37°C with 5% CO_2_ and protected from light for 30 min. Then, 2 mL FACS buffer (2,5 g bovine serum albumine in 500 mL phosphate-buffered saline) were added and samples were centrifuged at 1,500 RPM for 7 min at room temperature. After removal of the supernatant cells were resuspended in 200 μL FACS buffer and 40 μL were transferred to new polystyrene FACS tubes. These samples then were incubated with *Escherichia coli* (K-12 strain) BioParticles™ (Cat. nr.: #E-2861, Thermo Fisher, Waltham, USA), *Staphylococcus aureus* (Wood strain without protein A) BioParticles™ (Cat. nr.: #S-2851, Thermo Fisher), *Zymosan A S. cerevisiae* BioParticles™ (Cat. nr.: #Z-2841, Thermo Fisher) or nothing serving as controls at 37°C with 5% CO_2_ for 30 min and protected from light according to the manufacturer's instructions. Approximately 10 bioparticles per leukocyte were added and all were labeled with fluorescein isothiocyanate (FITC). Subsequently 2 mL FACS buffer were added and samples were centrifuged at 1,500 RPM for 5 min at room temperature followed by incubation in 0.5 mL of BD FACS Lysing Solution at room temperature and protected from light for 10 min. Then, 2 mL of FACS buffer was added and samples were centrifuged at 1,500 RPM for 7 min at room temperature. This step was repeated one more time and cells were diluted in 90 μL FACS buffer and stored on ice until measurement. From each sample a minimum of 50.000 cells was measured, which were subsequently analyzed. Monocyte and granulocyte populations were defined by gating the corresponding forward and side scatter scan and the duplets were excluded by plotting the height against the area for forward scatter. The percentage and the mean fluorescent units of FITC-labeled bioparticles engulfed by monocytes or granulocytes were assessed. The measurement was performed by flow cytometric analyses using a BD FACS Canto 2™ and FACS DIVA™ software (BD Biosciences, Franklin Lakes, USA).

### Statistical Analyses

All analyses were performed using GraphPad Prism 6 (Graphpad Software Inc., San Diego, USA). Data are presented as mean ± standard error of the mean. Based on the D'Agostino-Pearson normality test differences between the groups were determined by the non-parametric Kruskal-Wallis test followed by Dunn's *post hoc* test for the correction of multiple comparison. A *p*-value <0.05 was considered to be statistically significant.

## Results

### Granulocytes and Monocytes in Peripheral Blood

**Figure 2:**
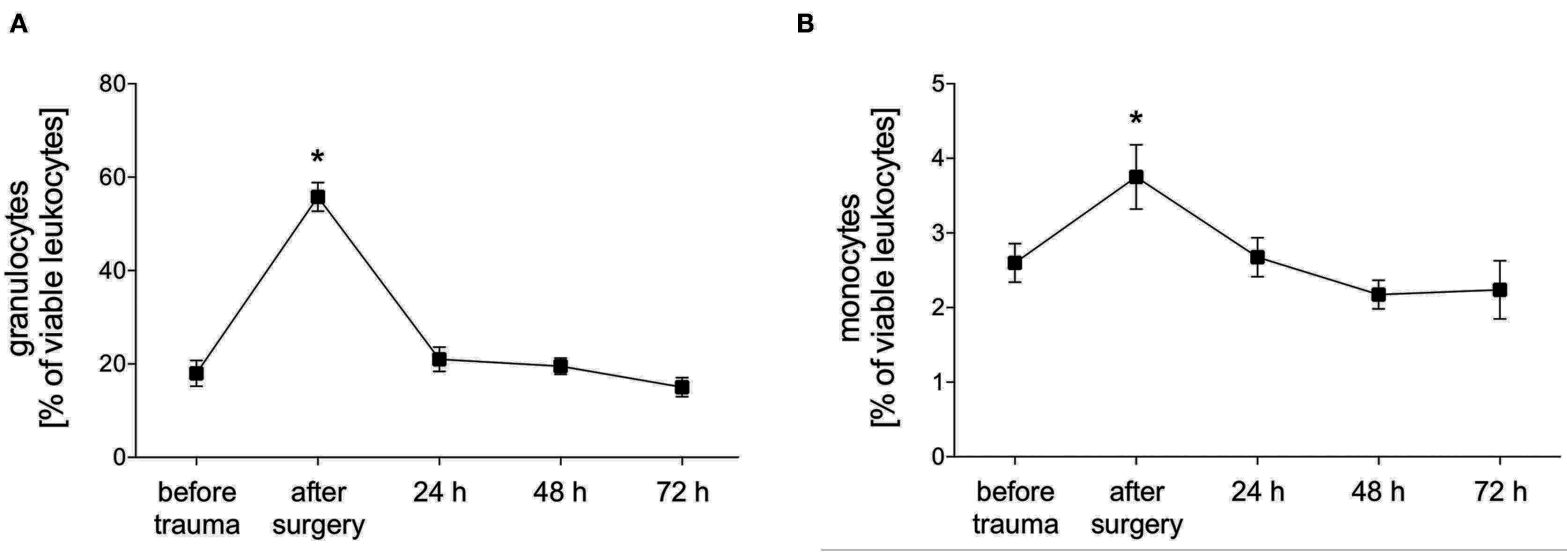
Percentage of granulocytes and monocytes of viable leukocytes. The percentage of granulocytes **(A)** and monocytes **(B)** of viable leukocytes was measured with FACS-analysis before trauma, after trauma and after 24, 48 and 72 h. Data are given as mean ± standard error of the mean, *p < 0.05 vs. baseline, *n* = 8.

The total proportion of granulocytes and monocytes out of viable leukocytes in peripheral blood was measured before trauma, after trauma and after 24, 48, and 72 h. Both granulocytes and monocytes showed a significant polytrauma-induced increase after surgery which declined to initial values again after 24 h (Figure 2).

### Phagocytizing Granulocytes and Their Phagocytic Capacity After Polytrauma

**Figure 3:**
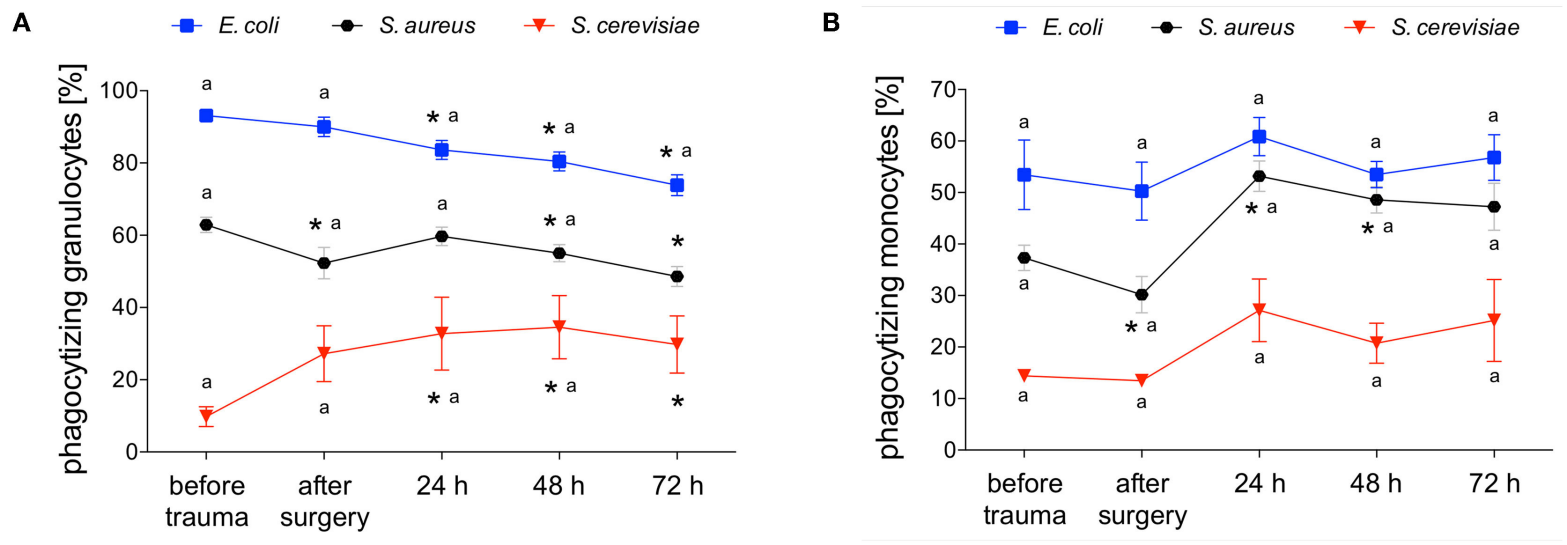
Percentage of *E. coli*-, *S. aureus*- or *S. cerevisiae*-phagocytizing granulocytes and monocytes. The percentage of *E. coli*-, *S. aureus*- or *S. cerevisiae*-phagocytizing granulocytes **(A)** and monocytes **(B)** was measured with FACS-analysis before trauma, after trauma and after 24, 48 and 72 h. Data are given as mean ± standard error of the mean, *p < 0.05 vs. baseline, *n* = 8.

**Figure 4:**
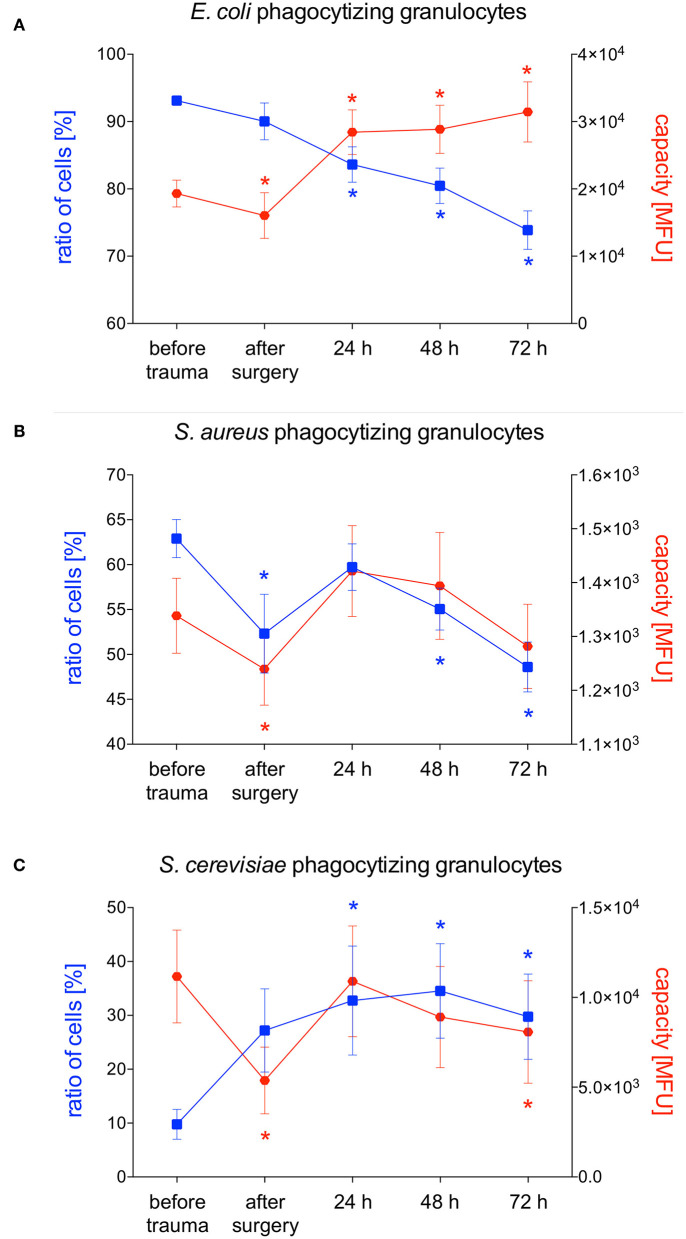
Percentage of phagocytizing granulocytes and phagocytic capacity for *E. coli, S. aureus* or *S. cerevisiae*. The percentage of phagocytizing granulocytes and their capacity to phagocytize *E. coli* **(A)**, *S. aureus* **(B)** or *S. cerevisiae* **(C)** was measured with FACS-analysis before trauma, after trauma and after 24, 48 and 72 h. Data are given as mean ± standard error of the mean, *p < 0.05 vs. baseline, *n* = 8.

*E. coli*-phagocytizing granulocytes significantly decreased after polytrauma and during further intensive care treatment. The capacity of granulocytes to phagocytize *E. coli* significantly increased in parallel during the intensive care treatment (Figures 3A, 4A). Interestingly granulocytes showed a different phagocytosis behavior for *S. aureus*. Both phagocytic activity and capacity of *S. aureus* significantly decreased after trauma. After 24 h a complete recovery was observed which was followed by another significant decrease, in both the number of phagocytizing granulocytes as well as their phagocytic capacity, after 48 and 72 h (Figures 3A, 4B). The percentage of *S. cerevisiae*-phagocytizing granulocytes significantly increased after 24 h and for the rest of the observation period. In contrast, a significant decrease of the *S. cerevisiae* phagocytic capacity was observed after surgery and 72 h (Figures 3A, 4C).

### Phagocytizing Monocytes and Their Phagocytic Capacity After Polytrauma

**Figure 5:**
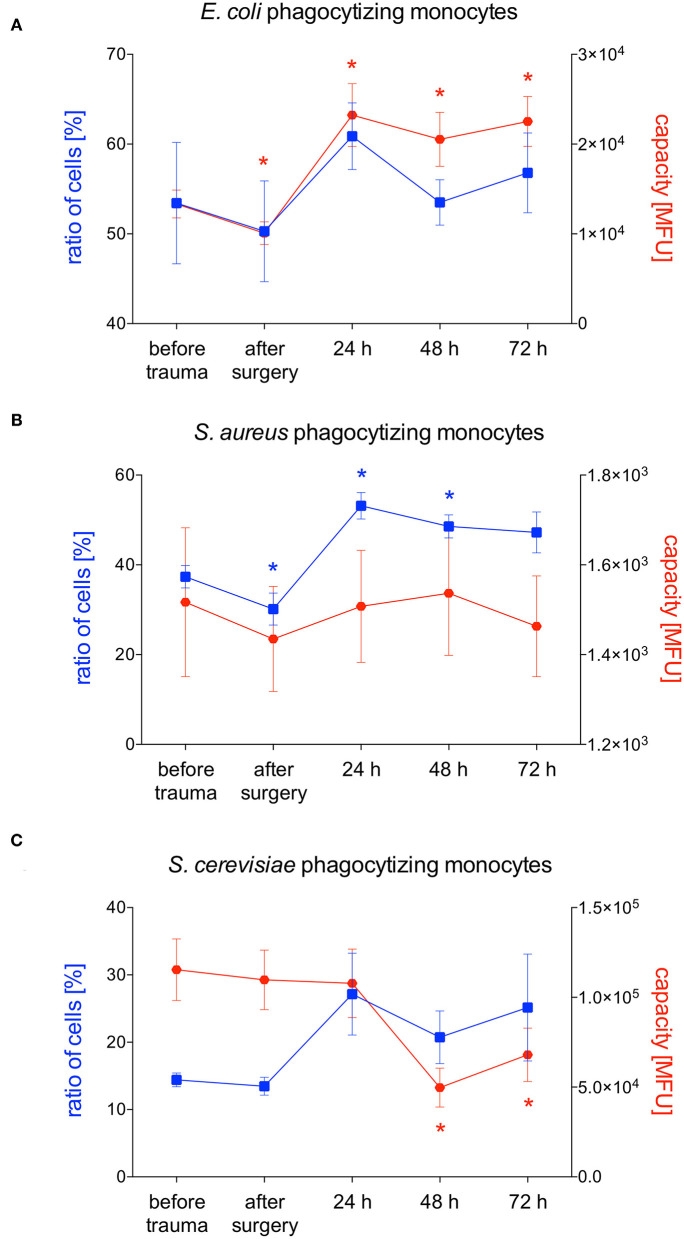
Percentage of phagocytizing monocytes and phagocytic capacity for *E. coli, S. aureus* or *S. cerevisiae*. The percentage of phagocytizing monocytes and their capacity to phagocytize *E. coli* **(A)**, *S. aureus* **(B)** *S. cerevisiae* **(C)** was measured with FACS-analysis before trauma, after trauma and after 24, 48 and 72 h. Data are given as mean ± standard error of the mean, *p < 0.05 vs. baseline, *n* = 8.

*E. coli*-phagocytizing monocytes did not change during the observation period while their capacity to phagocytize *E. coli* significantly decreases after surgery followed by a distinct and significant increase after 24 and 48 h (Figures 3B, 5A). The number of *S. aureus*-phagocytizing monocytes shows a significant decrease after surgery followed by a significant increase which was higher after 24 and 48 h compared to the initial values. The capacity of monocytes to phagocytize *S. aureus* did not change during the first three days after polytrauma (Figures 3B, 5B). The percentage of *S. cerevisiae*-phagocytizing monocytes did not show any significant changes during the study period while the capacity of monocytes to phagocytize *S. cerevisiae* significantly dropped after 48 and 72 h (Figures 3B, 5C).

## Discussion

Polytraumatized patients who survive the initial phase after injury remain at high risk to die from late-occurring infectious complications like sepsis, organ or multiple organ failure (MOF) (2, 3, 25, 26). Regardless of an infection or septic complication, trauma itself leads to a leukocytosis (27). We observed early leukocytosis in our porcine trauma model showing a significant peak of the number of granulocytes and monocytes immediately after surgery (Figures 2A,B). After 24 h in both cell types the levels turned to normal again possibly due to emigration of the leukocytes to the damaged tissue. Disturbed phagocytosis of neutrophils and/or monocytes has been linked to development of posttraumatic or postoperative sepsis (16). Therefore, we investigated phagocytic activity and capacity of granulocytes and monocytes in a standardized porcine polytrauma model during a prolonged observational period of 72 h after trauma. Interestingly, the phagocytic activity and capacity showed significantly different alterations depending on the pathogen strain (*E. coli, S. aureus* or *S. cerevisiae*).

*S. aureus* is one of the most common causative pathogens of hospital-acquired pneumonia (HAP)/ventilator-acquired pneumonia (VAP) and wound infections (12, 28). In our study, we observed a distinct and significant decrease in the total number of *S. aureus*-phagocytizing granulocytes after 48 and 72 h while their capacity to phagocytize *S. aureus* did not increase. Since granulocytes are not able to clear the bacterial load it remains to be clarified in further studies if a decreased number of *S. aureus*-phagocytizing granulocytes post-trauma may be a risk factor for developing pneumonia or wound infections. In contrast to our results it was shown that the percentage of *S. aureus*-phagocytizing granulocytes in polytraumatized patients increased significantly from day two till the end of the study period (10 days) (18). After severe injury granulocytes are replaced through (immature) bone marrow granulocyte which are rapidly released to the blood stream (29). This process is known as ‘emergency myelopoiesis’ (29). A possible reason for the initially decreased number of *S. aureus*-phagocytizing granulocytes observed in our study therefore might be their maturation state as immature granulocytes may not be able to perform phagocytosis. However, due to a study period of 72 h we can only speculate whether the number of *S. aureus*-phagocytizing granulocytes will return to “normal” or even higher numbers after three days or later will further decrease. Possibly we would also observe the same increase after three days that Sturm et al. already observed after two days. In line with our results phagocytic activity of granulocytes was impaired three days after traumatic brain injury (TBI) in mice (8). The authors also investigated the long term effect after TBI and showed impaired phagocytic activity of granulocytes still after 60 days (8). However, despite differences in immune response among different species, the posttraumatic response after brain injury and non-brain injury are not completely comparable. To date the changes in phagocytic behavior of granulocytes and monocytes after polytrauma are not completely characterized, otherwise it would be tempting to speculate about therapeutic options. Decreased phagocytic activity might be restored by medications activating phagocytosis (30–32). On the other hand, overwhelming phagocytosis might be reduced by inhibiting medications (33, 34). However, septic complications after trauma do not necessarily have to be caused by reduced or overstimulated phagocytosis itself. Phagosomal acidification, another critical part of neutrophil function, has been shown to be altered in polytraumatized patients developing septic complications (35). Nevertheless, it must be mentioned that these investigations have been made in a relatively small patient cohort of only 15 patients (35).

In our study, we observed early decreased phagocytic activity of monocytes for *S. aureus* while phagocytic activity for *E. coli* and *S. cerevisiae* were not altered. Schimunek et al. investigated the number of phagocytes as well as their capacity to phagocytize *S. aureus* in the porcine polytrauma model. In line with our results, an early decreased phagocytic activity of monocytes was observed (19). Furthermore, the authors showed reduced phagocytic capability for *S. aureus* after surgery (19). Both the phagocytic activity and capability recovered to baseline after 24 h (19). In the underlying study, we also observed a significant decrease of monocytic phagocytic activity after surgery but in contrast to the study by Schimunek et al. the phagocytic activity increased to higher values compared to the baseline after 24 and 48 h. In line with this a significantly increased phagocytic activity of monocytes in mice 3 days after TBI was reported (8). In polytraumatized patients the number of *S. aureus* phagocytizing monocytes was also diminished early after trauma and recovered to baseline after 2 days (18). Obviously, the majority of studies report early decreased monocytic phagocytic activity followed by return to normal or even increased activity. Possibly after trauma monocytes leave blood stream by migrating into the traumatized tissue and are replaced by immature cells from the bone marrow that need time to bring phagocytic activity to “normal” or even higher state again. The capacity to phagocytize *S. aureus* did not significantly change in our study but we observed an overall higher rate in the phagocytic capacity of *S. aureus* compared to Schimunek et al. (19). Possible differences may have been observed due to the use of heparin blood samples instead of EDTA blood in the underlying study as compared to Schimunek et al. (36). In contrast, Seshadri et al. incubated monocytes with (FITC)-labeled *S. aureus* and opsonizing reagent to evaluate phagocytosis and did not see any changes in monocyte phagocytosis after injury (7). Pérez-Bárcena et al. compared TLR2 and TLR4 functionality in trauma patients receiving nutrition with or without glutamine supplement as use of glutamine as a dietary supplement is associated with a reduced risk of infection and observed no changes in phagocytic capability of monocytes between the two groups (37).

Besides pneumonia, infections of the urinary tract are a common complication during clinical course after trauma (12). These infections are often caused by *E. coli* which is why we also investigated the phagocytic activity and capacity of granulocytes and monocytes for *E. coli* in the underlying porcine trauma model. Interestingly we observed a different pattern than for phagocytosis of *S. aureus*. The percentage of *E. coli*-phagocytizing granulocytes significantly decreased early after trauma with a continuous decrease till the end of the study period. Probably as a compensatory mechanism the capacity of granulocytes significantly increased after 24 h, thus, among several others, maybe giving a gentle hint why infections with *E. coli* are less frequent than infections with *S. aureus*. In line with our results Liao et al. observed significantly decreased percentage of *E. coli*-phagocytizing granulocytes at 24, 48 and 72 h in trauma patients compared to healthy controls (38). Furthermore, in TBI patients *E. coli*-phagocytizing activity of granulocytes was decreased during the whole study period of 2 weeks (38). No difference in the percentage of neutrophils able to do phagocytosis but a significantly lower ability of neutrophils to phagocytose fluorescent *E. coli* were observed in patients with spinal cord injury (39). The above mentioned decline in phagocytosis has been suggested to represent a compensatory mechanism, where reduced phagocytosis offsets the deleterious effects that the increase in ROS generation, that has been described especially in patients with TBI and SCI but also with blunt or penetrating trauma, would have on bystander tissue (38, 40). Nevertheless, given that granulocytes are the first-line of defense against invading microbes, a reduced phagocytosis may also be detrimental to the host and therefore at least in part explains the high incidence of nosocomial infections reported amongst patients following neurological trauma (40, 41). In line with these observations, we also observed reduced phagocytic activity of granulocytes for *S. aureus* and *E. coli*, two pathogens frequently seen in nosocomial infections. Furthermore, a reduced phagocytosis of neutrophils in the first 24 h of sepsis and a reduced phagocytic activity of granulocytes and monocytes in septic patients after trauma or surgery have been reported (15, 16). On the other hand, Spittler et al. compared the phagocytic capacity of monocytes from ten patients with sepsis with low IL-6 serum concentrations and eight patients with sepsis with high IL-6 plasma concentrations and observed significantly increased phagocytic properties as well as worse outcomes in patients with high IL-6 levels (17).

Invasive fungal infections are rare but serious complications of traumatic injury with an increasing incidence of approximately 0.43–1.7 cases per million persons (42). In line with our reported results for phagocytosis of *S. cerevisiae* granulocytes in the group of multiple traumatized patients showed significantly higher yeast-phagocytizing activity on the first and third day after injury compared to the control group (43). This higher phagocytic activity of granulocytes might, among others, be a reason for the lower frequency of invasive fungal infections.

In this study, we observed significant changes in phagocytic activity and capacity of granulocytes and monocytes after polytrauma. It still has to be further elucidated whether increased phagocytic activity or capacity is a “beneficial” inflammatory state and if their reduced phagocytic activity, as seen for *S. aureus* and *E. coli*, puts polytraumatized patients at risk to developing septic complications. On the other hand, reduced phagocytic activity might also be a “beneficial” mechanism to reduce overwhelming inflammatory response which would lead to further tissue damage. In future studies it remains important to investigate the phagocytic behavior of leukocytes which have emigrated from blood to tissue since it has been shown that the surrounding milieu has an impact on the phagocytic behavior (43). This study has several limitations of which the most important one is the limited sample size. Moreover, it remains to be elaborated, if the above described changes in phagocytic activity and capacity will influence the outcome after trauma beyond the observational period of 72 h, as our findings cannot be linked to defined outcomes like infections, sepsis, length of stay on ICU or requirement for ventilation. The interspecies variability in innate immune response has to be taken into consideration when results of animal experiments are transferred to human situation (44). Furthermore, no data of samples without PMA-stimulation could be provided.

Taken together, our data indicate that phagocytic activity and capacity of granulocytes and monocytes follow a different pattern and significantly change within 72 h after polytrauma. Furthermore, the phagocytic activity and capacity show significantly different alterations depending on the pathogen strain.

## Data Availability Statement

The original contributions generated for this study are included in the article/supplementary material, further inquiries can be directed to the corresponding author/s.

## Ethics Statement

Experiments were authorized by the responsible government authority (Landesamt für Natur-, Umwelt- und Verbraucherschutz: LANUV-NRW, Germany). The approval number is: AZ: 81.02.04.2018.A113.

## Author Contributions

BR: conceptualization and supervision. JV, FK, FB, JG, EB, AJ, ID, and BR: methodology. JV: validation, data curation, and writing-original draft preparation. BR and JV: formal analysis and visualization. JV, FB, JG, and EB: investigation. JV, FK, FB, JG, EB, AJ, ID, FH, IM, and BR: writing-review and editing. JV, FB, and BR: project administration. BR and FH: funding acquisition. All authors: contributed to the article and approved the submitted version.

## Conflict of Interest

The authors declare that the research was conducted in the absence of any commercial or financial relationships that could be construed as a potential conflict of interest.

## References

[1] World Health Organization. Global Health Estimates 2016: Disease Burden by Cause, Age, Sex, by Country and by Region, 2000-2016. Geneva: World Health Organization (2018).

[2] Nast-Kolb D Aufmkolk M Rucholtz S Obertacke U Waydhas C. Multiple organ failure still a major cause of morbidity but not mortality in blunt multiple trauma. J Trauma. (2001) 51:835–41. 10.1097/00005373-200111000-00003 11706328

[3] Relja B Land WG. Damage-associated molecular patterns in trauma. Eur J Trauma Emerg Surg. (2019) 324:1–25. 10.1007/s00068-019-01235-w PMC7427761 31612270

[4] Dutton RP Stansbury LG Leone S Kramer E Hess JR Scalea TM. Trauma mortality in mature trauma systems: are we doing better? An analysis of trauma mortality patterns, 1997-2008. J Trauma. (2010) 69:620–6. 10.1097/TA.0b013e3181bbfe2a 20093983

[5] Gerber LM Chiu Y-L Carney N Härtl R Ghajar J. Marked reduction in mortality in patients with severe traumatic brain injury. J Neurosurg. (2013) 119:1583–90. 10.3171/2013.8.JNS13276 24098983

[6] Osborn TM Tracy JK Dunne JR Pasquale M Napolitano LM. Epidemiology of sepsis in patients with traumatic injury. Crit Care Med. (2004) 32:2234–40. 10.1097/01.CCM.0000145586.23276.0F 15640635

[7] Seshadri A Brat GA Yorkgitis BK Keegan J Dolan J Salim A. Phenotyping the immune response to trauma. Crit Care Med. (2017) 45:1523–30. 10.1097/CCM.0000000000002577 28671900 PMC10114604

[8] Ritzel RM Doran SJ Barrett JP Henry RJ Ma EL Faden AI. Chronic alterations in systemic immune function after traumatic brain injury. J Neurotrauma. (2018) 35:1419–36. 10.1089/neu.2017.5399 29421977 PMC5998829

[9] Kolaczkowska E Kubes P. Neutrophil recruitment and function in health and inflammation. Nat Rev Immunol. (2013) 13:159–75. 10.1038/nri3399 23435331

[10] Winterbourn CC Kettle AJ Hampton MB. Reactive oxygen species and neutrophil function. Annu Rev Biochem. (2016) 85:765–92. 10.1146/annurev-biochem-060815-014442 27050287

[11] Lambert C Preijers FWMB Yanikkaya Demirel G Sack U. Monocytes and macrophages in flow: an ESCCA initiative on advanced analyses of monocyte lineage using flow cytometry. Cytometry B Clin Cytom. (2017) 92:180–8. 10.1002/cyto.b.21280 26332381

[12] Kerwat K Graf J Wulf H. Krankenhaushygiene – nosokomiale infektionen. Anästhesiol Intensivmed Notfallmed Schmerzther. (2010) 45:30–1. 10.1055/s-0029-1243375 20091478

[13] Papia G McLellan BA El-Helou P Louie M Rachlis A Szalai JP. Infection in hospitalized trauma patients: incidence, risk factors, and complications. J Trauma. (1999) 47:923–7. 10.1097/00005373-199911000-00018 10568723

[14] Flannagan RS Jaumouillé V Grinstein S. The cell biology of phagocytosis. Annu Rev Pathol. (2012) 7:61–98. 10.1146/annurev-pathol-011811-132445 21910624

[15] Danikas DD Karakantza M Theodorou GL Sakellaropoulos GC Gogos CA. Prognostic value of phagocytic activity of neutrophils and monocytes in sepsis. Correlation to CD64 and CD14 antigen expression. Clin. Exp. Immunol. (2008) 154:87–97. 10.1111/j.1365-2249.2008.03737.x 18727624 PMC2561092

[16] Hirsh M Mahamid E Bashenko Y Hirsh I Krausz MM. Overexpression of the high-affinity Fcgamma receptor (CD64) is associated with leukocyte dysfunction in sepsis. Shock. (2001) 16:102–8. 10.1097/00024382-200116020-00003 11508860

[17] Spittler A Razenberger M Kupper H Kaul M Hackl W Boltz-Nitulescu G. Relationship between interleukin-6 plasma concentration in patients with sepsis, monocyte phenotype, monocyte phagocytic properties, and cytokine production. Clin Infect Dis. (2000) 31:1338–42. 10.1086/317499 11095999

[18] Sturm R Heftrig D Mörs K Wagner N Kontradowitz K Jurida K. Phagocytizing activity of PMN from severe trauma patients in different post-traumatic phases during the 10-days post-injury course. Immunobiology. (2017) 222:301–7. 10.1016/j.imbio.2016.09.010 27745899

[19] Schimunek L Serve R Teuben MPJ Störmann P Auner B Woschek M. Early decreased TLR2 expression on monocytes is associated with their reduced phagocytic activity and impaired maturation in a porcine polytrauma model. PLoS ONE. (2017) 12:e0187404. 10.1371/journal.pone.0187404 29125848 PMC5681268

[20] National Research Council (US) Committee for the Update of the Guide for the Care and Use of Laboratory Animals. Guide for the Care and Use of Laboratory Animals. Washington, DC: National Academies Press (2011). 21595115

[21] Kilkenny C Browne WJ Cuthill IC Emerson M Altman DG. Improving bioscience research reporting: the ARRIVE guidelines for reporting animal research. Osteoarthr Cartil. (2012) 20:256–60. 10.1016/j.joca.2012.02.010 22424462

[22] Horst K Simon TP Pfeifer R Teuben M Almahmoud K Zhi Q. Characterization of blunt chest trauma in a long-term porcine model of severe multiple trauma. Sci Rep. (2016) 6:39659. 10.1038/srep39659 28000769 PMC5175194

[23] ATLS Subcommittee, American College of Surgeons' Committee on Trauma, International ATLS working group. Advanced trauma life support (ATLS®): the ninth edition. J Trauma Acute Care Surg. (2013) 74:1363–6. 10.1097/01586154-201305000-00026 23609291

[24] Nikolaou NI Welsford M Beygui F Bossaert L Ghaemmaghami C Nonogi H. Part 5: Acute coronary syndromes: 2015 International consensus on cardiopulmonary resuscitation and emergency cardiovascular care science with treatment recommendations. Resuscitation. (2015) 95:e121–46. 10.1016/j.resuscitation.2015.07.043 26477704

[25] Pfeifer R Tarkin IS Rocos B Pape H-C. Patterns of mortality and causes of death in polytrauma patients–has anything changed? Injury. (2009) 40:907–11. 10.1016/j.injury.2009.05.006 19540488

[26] Relja B Mörs K Marzi I. Danger signals in trauma. Eur J Trauma Emerg Surg. (2018) 44:301–16. 10.1007/s00068-018-0962-3 29728738 PMC6002466

[27] Riley LK Rupert J. Evaluation of patients with leukocytosis. Am Fam Physician. (2015) 92:1004–11. 26760415

[28] Barbier F Andremont A Wolff M Bouadma L. Hospital-acquired pneumonia and ventilator-associated pneumonia. Curr Opin Pulm Med. (2013) 19:216–28. 10.1097/MCP.0b013e32835f27be 23524477

[29] Nacionales DC Szpila B Ungaro R Lopez MC Zhang J Gentile LF. A detailed characterization of the dysfunctional immunity and abnormal myelopoiesis induced by severe shock and trauma in the aged. J Immunol. (2015) 195:2396–407. 10.4049/jimmunol.1500984 26246141 PMC4546902

[30] Alsharif KF Thomas MR Judge HM Khan H Prince LR Sabroe I. Ticagrelor potentiates adenosine-induced stimulation of neutrophil chemotaxis and phagocytosis. Vascul Pharmacol. (2015) 71:201–7. 10.1016/j.vph.2015.02.006 25869515 PMC4534709

[31] Chen M-L Wu S Tsai T-C Wang L-K Tsai F-M. Regulation of neutrophil phagocytosis of *Escherichia coli* by antipsychotic drugs. Int Immunopharmacol. (2014) 23:550–7. 10.1016/j.intimp.2014.09.030 25448498

[32] Pinder EM Rostron AJ Hellyer TP Ruchaud-Sparagano M-H Scott J Macfarlane JG. Randomised controlled trial of GM-CSF in critically ill patients with impaired neutrophil phagocytosis. Thorax. (2018) 73:918–25. 10.1136/thoraxjnl-2017-211323 30064991 PMC6166597

[33] Mancuso P Nana-Sinkam P Peters-Golden M. Leukotriene B4 augments neutrophil phagocytosis of Klebsiella pneumoniae. Infect Immun. (2001) 69:2011–6. 10.1128/IAI.69.4.2011-2016.2001 11254552 PMC98124

[34] Ries F Alflen A Aranda Lopez P Beckert H Theobald M Schild H. Antifungal drugs influence neutrophil effector functions. Antimicrob Agents Chemother. (2019) 63:1435. 10.1128/AAC.02409-18 30910895 PMC6535511

[35] Hesselink L Spijkerman R de Fraiture E Bongers S Van Wessem KJP Vrisekoop N. New automated analysis to monitor neutrophil function point-of-care in the intensive care unit after trauma. Intensive Care Med Exp. (2020) 8:12–12. 10.1186/s40635-020-0299-1 32172430 PMC7072076

[36] Ducusin RJ Sarashina T Uzuka Y Tanabe S Ohtani M. Phagocytic response of bovine polymorphonuclear leukocytes to different incubation conditions and following exposure to some effectors of phagocytosis and different anticoagulants *in vitro*. Can J Vet Res. (2001) 65:38–44. 11227193 PMC1189640

[37] Perez-Barcena J Crespi C Regueiro V Marsé P Raurich JM Ibáñez J. Lack of effect of glutamine administration to boost the innate immune system response in trauma patients in the intensive care unit. Crit Care. (2010) 14:1–11. 10.1186/cc9388 21184675 PMC3219991

[38] Liao Y Liu P Guo F Zhang Z-Y Zhang Z. Oxidative burst of circulating neutrophils following traumatic brain injury in human. PLoS ONE. (2013) 8:e68963–12. 10.1371/journal.pone.0068963 23894384 PMC3722225

[39] Kanyilmaz S Hepguler S Atamaz FC Gokmen NM Ardeniz O Sin A. Phagocytic and oxidative burst activity of neutrophils in patients with spinal cord injury. Arch Phys Med Rehabil. (2013) 94:369–74. 10.1016/j.apmr.2012.09.015 23022452

[40] Hazeldine J Hampson P Lord JM. The impact of trauma on neutrophil function. Injury. (2014) 45:1824–33. 10.1016/j.injury.2014.06.021 25106876

[41] Boque MC Bodi M Rello J. Trauma, head injury, and neurosurgery infections. Semin Respir Infect. (2000) 15:280–6. 10.1053/srin.2000.20935 11220410

[42] Kronen R Liang SY Bochicchio G Bochicchio K Powderly WG Spec A. Invasive fungal infections secondary to traumatic injury. Int J Infect Dis. (2017) 62:102–11. 10.1016/j.ijid.2017.07.002 28705753

[43] Pap G Furész J Fennt J Kovács GC Nagy L Hamar J. Self-regulation of neutrophils during phagocytosis is modified after severe tissue injury. Int J Mol Med. (2006) 17:649–54. 10.3892/ijmm.17.4.649 16525723

[44] Matute-Bello G Frevert CW Martin TR. Animal models of acute lung injury. Am J Physiol Lung Cell Mol Physiol. (2008) 295:L379–99. 10.1152/ajplung.00010.2008 18621912 PMC2536793

